# Pressurized Hot Water Extraction as Green Technology for Natural Products as Key Technology with Regard to Hydrodistillation and Solid–Liquid Extraction

**DOI:** 10.1021/acsomega.4c03771

**Published:** 2024-07-10

**Authors:** Larissa Knierim, Alexander Uhl, Axel Schmidt, Marcel Flemming, Theresa Höß, Jonas Treutwein, Jochen Strube

**Affiliations:** † Institute for Separation and Process Technology, Clausthal University of Technology, D-38678 Clausthal-Zellerfeld, Germany; ‡ SKH GmbH, Koenigbacher Strasse 17, D-94496 Ortenburg, Germany; § Trifolio-M GmbH, Dr.-Hans-Wilhelmi-Weg 1, D-35633 Lahnau, Germany

## Abstract

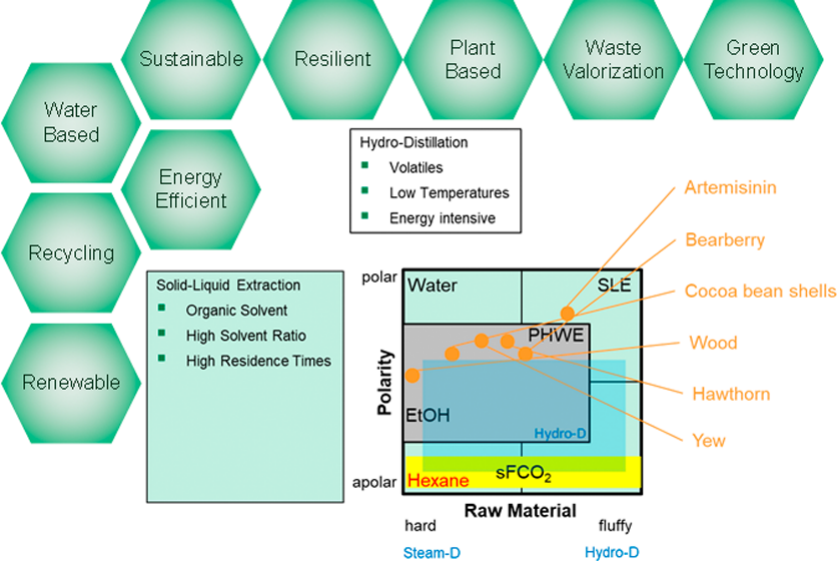

Hydrodistillation and solid–liquid extraction with organic solvents or supercritical CO_2_ are standard technologies for natural product manufacturing. Within this technology, portfolio pressurized hot water technology is ranked as a green, sustainable, resilient, kosher and halal manufacturing process. Essential for sustainability is energy integration for heating and cooling the auxiliary water as well as product concentration without evaporation but with the aid of low energy consuming ultra- and nanofiltration membrane technology. The incorporation of modern unit operations, such as pressurized hot water extraction, along with inline measurement devices for Process Analytical Technology approaches, showcases a shift in traditional extraction processes. Traditional equipment and processes still dominate the manufacturing of plant extracts, yet leveraging innovative chemical process engineering methods offers promising avenues for the economic and ecological advancement of botanicals. Techniques such as modeling and process intensification with green technology hold potential in this regard. Digitalization and Industry 4.0 methodologies, including machine learning and artificial intelligence, play pivotal roles in sustaining natural product extraction manufacturing and can profoundly impact the future of human health.

## Introduction

**Figure 1:**
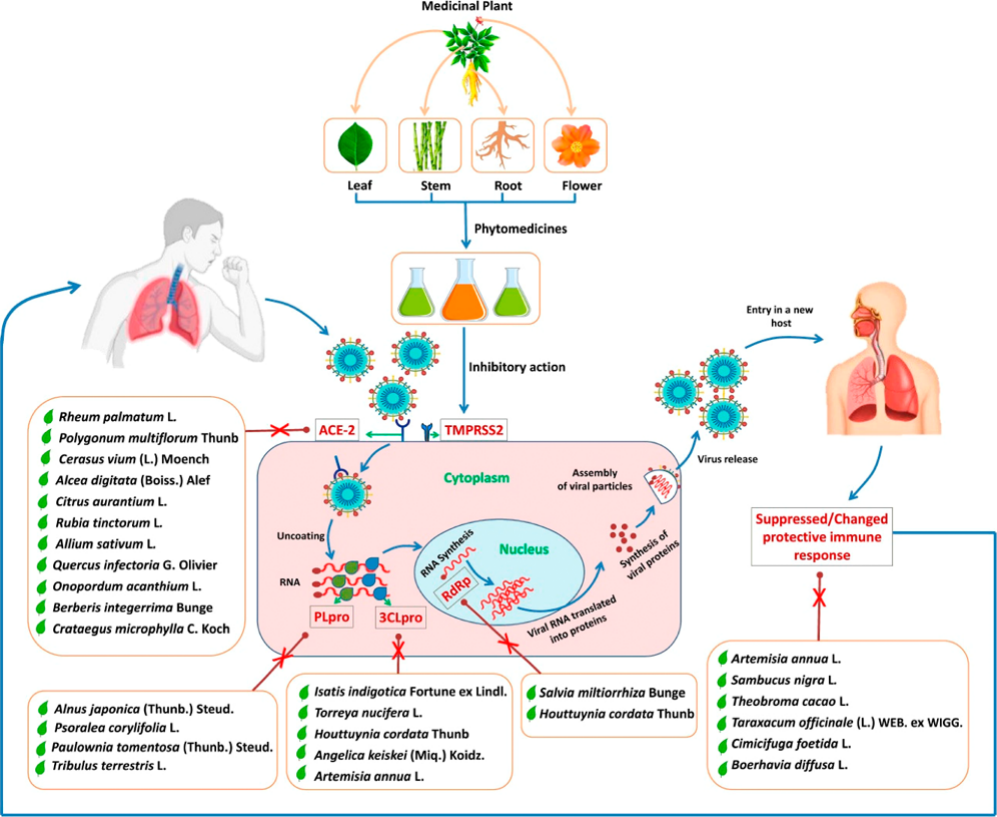
Herbal medicines for the treatment of respiratory diseases. Reprinted with permission from ref (5). Copyright 2020 Siddiqui.

The use of plants to treat a wide range of diseases has been practiced for thousands of years. Currently, around 26% of authorized medicines are of plant origin.^1^ Of the 300,000 plants known worldwide, 10,000 have a proven medicinal effect, although only 150–200 of these are used in western medicine to treat diseases.^2,3^ The advantage of herbal medicines is that they are already naturally integrated into the biological cycle and are therefore biodegradable. The spectrum in which phytopharmaceuticals are used ranges from cardiovascular diseases, cancer with 10-DAB, diabetes, antibiotics, malaria with artemisinin, and various combination preparations.^2,4^ During the corona pandemic, a wide variety of herbal medicines were used to support the healing of respiratory diseases. Examples and their mode of action are shown in Figure 1.^5^

Furthermore, adjuvants are used in vaccines that are also of plant origin, such as QS-21 from the soap bark tree for the formulation of lipid nanoparticle vaccines.^6^

As the land must be used primarily for the production of food, it should be decided exactly which other plants can be grown there to have the same added value for society.^7^ Therefore, the only option here is to utilize waste streams generated during the production of food. The processes used to utilize these should also be sustainable and, ideally, water-based. The challenge here is the regulatory restrictions that must be observed in the production of food, food supplements, and cosmetics and, above all, in the production of phytopharmaceuticals. Hydrodistillation for the selective extraction of essential oils and pressurized hot water extraction (PHWE) for the extraction of secondary metabolites, fibers, lignins, cellulose, and hemicellulose can be used as a water-based cascade utilization of side streams. However, the processes used should not only reduce the global warming potential (GWP), but also have lower cost of goods (COGs) so that the investments made are also economically viable.

Currently, many plant extraction processes are still based on toxic solvents and multistep processes in which little consideration is given to energy and chemical consumption and in which waste streams accumulate because they are not reused.^8,9^ When developing new processes, waste streams should be avoided as far as possible, and safe solvents and process conditions should be used. The processes should also have high energy efficiency and be based on renewable feedstocks. Real-time analyses should also be integrated to avoid environmental pollution, accidents, and batch failure. Potentially hazardous substances must be avoided, and the long-term effects of components must be taken into account. The processes should involve as few steps as possible to reduce energy and chemical consumption and avoid waste streams.^10^

The alternative solvents that can be used for this are, for example, supercritical CO_2_, ionic or eutectic mixtures, fluorinated solvents, liquid polymers, and green solvents such as bioethanol or other biosolvents.^11^ The PHWE alternative considered here uses subcritical water for extraction. The advantages here are that water is a cheap and green solvent, which is also available in large quantities and is easy to purify.^12^

This work first deals with the fundamentals of PHWE and the application field. Subsequently, the influence of the process and material parameters is investigated with an already validated model using a new material system for which the optimal parameters are also determined. Afterward, the occurring phenomena of PHWE are compared to the ones in classical extraction methods.

## Fundamentals

**Figure 2:**
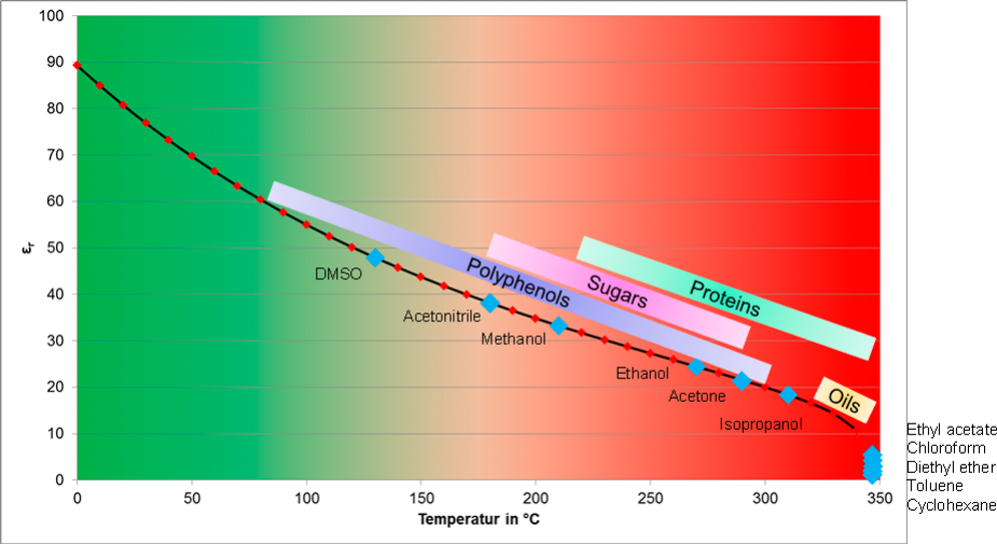
Change of water polarity with temperature as well as polarities of organic solvents and substance groups in comparison.

**Figure 3:**
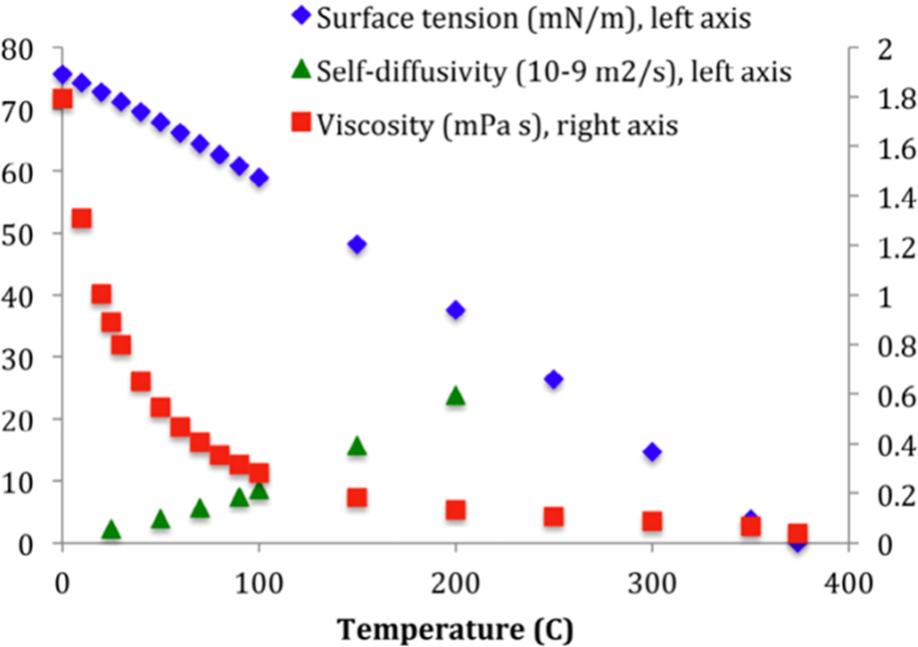
Change of water surface tension, self-diffusivity, and viscosity with temperature. Reprinted with permission from ref (13). Copyright 2015 Elsevier.

**Figure 4:**
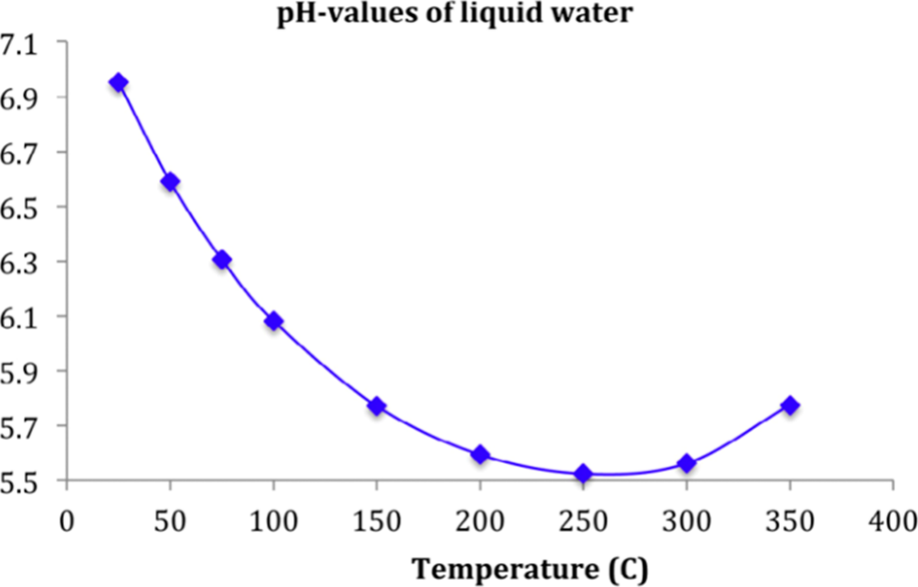
Change of pH value of water with temperature. Reprinted with permission from ref (13). Copyright 2015 Elsevier.

The way PHWE works is that water changes its physical properties as the temperature rises. The focus here is on the change in the polarity of the water, which is described by the dielectric constant. As the temperature rises, this shifts toward the nonpolar range and achieves similar values to methanol or ethanol, for example (Figure 2). This makes it possible to extract nonpolar components without using organic solvents. Furthermore, the surface tension, self-diffusivity, viscosity (Figure 3), and pH value of the water also change as the temperature rises (Figure 4).

**Figure 5:**
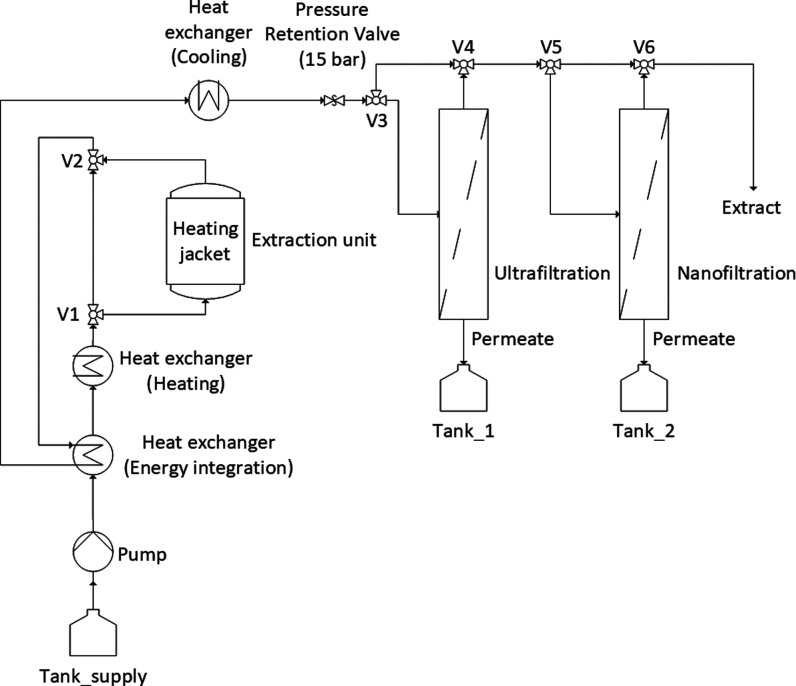
PHWE with energy integration as well as ultra- and nanofiltration for water recycling.

The PHWE is operated between the boiling point of water (100 °C at 1 bar) and the critical point (374 °C at 221 bar), usually at a temperature of 100–160 °C and a pressure of 2–12 bar. Because of high pressures and temperatures during extraction, special equipment and material are needed to provide safe execution. The process can be operated either as maceration in the form of an externally heated and pressurized boiler or as pressurized percolation, in which case the water is heated before entering the extraction column (Figure 5). The challenge here is the thermal decomposition of the target component. In the percolation mode, this can be counteracted with both the temperature and the residence time of the solvent in the column.

## Products

PHWE has a wide range of applications and can be used as an alternative to hydro or water vapor distillation for extracting essential oils from oregano, thyme, rose, rosemary, marjoram, savory, peppermint, clove, sage, and sideritis or for extracting carotenoids from green algae, carrots, green beans, and broccoli. The largest area of application is the extraction of phenolic components from, for example, winery byproducts, ginseng, tea, grape seeds, noni, orange peel, flaxseed (lignans), knotwood, olive leaves, potato peel, and berries.^12^ Further plant-based raw materials and target substances can be found in Table 1.

**Table 1:** Recent Studies of Phenols Extracted from Natural Resources Using PHWE^a^

raw material	target compound	optimal operating conditions	ref
mango peels	total phenols	180 °C, 90 min, solid to water ratio 1:40	(14)
uva ursi herbal dust	total phenols and total flavonoids	151.2 °C, 10 min, 1.5% HCl	(15)
wild garlic (*Allium ursinum* L.)	total phenols and total flavonoids	180.92 °C, 10 min, added acidifier 1.09%	(16)
white grape pomace	total phenols	210 °C, 100 bar, 30 min	(17)
winter savory (*Satureja montana* L.)	total phenols and total flavonoids	220 °C, 20.8 min, 30 bar	(18)
spent coffee grounds (*Coffea arabica* L.)	total phenols	177 °C, 55 min, 50 bar	(19)
ginger	gingerol	130 °C, 20 min, 2 bar	(20)
black tea	myrcetin and quercetin kaempherol	170 °C, 15 min, 101 bar, 200 °C, 15 min, 101 bar	(21)
celery powder	myrcetin and quercetin kaempherol	170 °C, 15 min, 101 bar, 200 °C, 15 min, 101 bar	(21)
ginseng leaf	myrcetin and quercetin kaempherol	170 °C, 15 min, 101 bar, 200 °C, 15 min, 101 bar	(21)
tumeric rhizomes (*Curcuma longa* L.)	curcumin	140 °C, 14 min, 10 bar	(22)

a Reproduced with permission from ref (11). Copyright 2019 Elsevier

In addition, extraction by PHWE can be used to determine pesticides and PAHs in nutrient products such as black tea and edible vegetable oils.^12^

A selection of plants and the target components extracted from them using PHWE is listed in Table 2.

**Table 2:** Target Components of Plants and Food Materials Extracted with PHWE^a^

analyte(s)	matrix	temperature (°C)	pressure	flow rate(mL/min)	extraction time (min)	ref
**Plants**						
stevioside, rebaudioside A	*Stevia rebaudiana*	100	11–13 bar	1.5	15	(24)
gastrodin, vanillyl alcohol	*Gastrodia elata*	100	8–10 bar	1.5	20	(25)
phenolic compounds	*Momordica charantia*	150–200	10 MPa	2.0	320	(26)
tanshinone I and IIA	*Salvia miltiorrhiza*	95–140	10–20 bar	1.0	20, 40	(27)
essential oil	*Fructus amomi*	150	50 bar	1.0	5	(28)
essential oil	*Acorus tatarinowii*	150	50 bar	1.0	5	(29)
essential oil	*Fructus amomi*	160	60 bar	1.0	5	(30)
borneol, terpinen-4-ol, carvacrol	*Origanum anites*	100, 125, 150, 175	60 bar	2.0	30	(31)
essential oils	*Origanum micrathum*	100, 125, 150, 175	40–80 bar	1.0–3.0	30	(32)
pulegone, terpinen-4-ol, *trans*-carveol, verbenone	*Ziziphora taurica*	150	60 bar	2.0	30	(33)
glycyrrhizin	*Glycyrrhiza glabra*	30–120	5 atm	nil	60–120	(34)
anthocyanins	*Brassica oleracea*	80–120	50 bar	nil	11	(35)
anthraquinones	*Morinda citrifolia*	80, 120	4 MPa	4.0	120	(36)
saponins, cyclopeptides	*Vaccaria segetalis* Garcke, *Saponaria vaccaria*	160	750 psi	0.5, 1.0, 2.0, 4.0, 8.0	80	(37)
terpenes (α-pinene, limonene, camphor, citronellol, carvacrol)	basil and oregano leaves	100, 150, 200, 250	nil	nil	30, 300	(38)
volatile oil	*Cuminum cyminum* L.	100–175	20 bar	2.0, 4.0	nil	(39)
lignans	*Linum usitatissimum*	140	5.2 MPa	0.5	400	(40)
rosmarinic acid, carnosic acid	*Rosmarinus officinalis*	60–100	1500 psi	nil	25	(41)
antioxidants	*Spirulina platensis*	60, 115, 170	1500 psi	nil	3, 9, 15	(42)
antioxidants	*Spirulina platensis*	115, 170	1500 psi	nil	9, 15	(43)
cedarwood oil	*Juniperus virginianna*	50, 100, 150, 200	500, 750, 1500, 3000 psi	nil	15	(44)
1,1-diphenyl-2-picrylhydrazyl	*Diascorea alata*	100	1.34 MPa	10.0	<180	(45)
anthraquinones	*Morinda citrifolia*	100, 170, 220	7 MPa	2.0, 4.0, 6.0	18	(46)
**Food**						
total sugars, proteins	defatted rice bran	200	nil	nil	5	(47)
isoflavones	soybeans	100	1000 psi	nil	nil	(48)
lignans, proteins, and carbohydrates	defatted flaxseed meal	130, 160, 190	750 psi	1.0	400	(49)
flavonoids	knotwood of aspen	150	220 bar	nil	35	(50)
catechins, proanthocyanidins	grape seed	50, 100, 150	1500 psi	nil	30	(51)
capsaicin, dihydrocapsaicin	peppers	50–200	100 atm	nil	nil	(52)
anthocyanins, phenolics	dried red grape skin	100–160	nil	nil	40 s	(53)
catechin, epicatechin	tea leaves, grape seeds	100–200	1500 psi	nil	5, 10	(54)
isoflavones	defatted soybean flakes	110	641 psig	nil	2.3 h	(55)
total phenolic content	citrus pomaces	25–250	0.1–5.0 MPa	nil	10, 30, 60	(56)

a Reproduced with permission from ref (23). Copyright 2010 Elsevier

**Figure 6:**
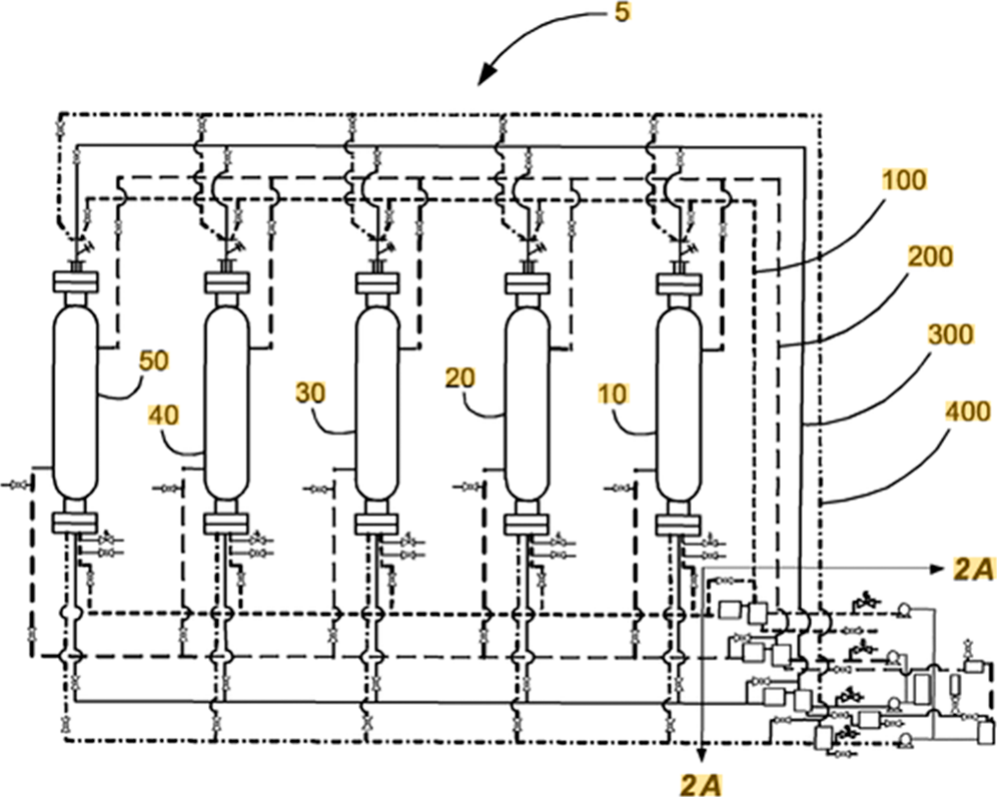
Multicolumn PHWE by Mazza Innovation. Reproduced with permission from ref (57). Copyright 2015 Mazza Innovation, Ltd.

The PHWE can be used not only as an extraction system in the form of one flow-through column but also as an interconnection of several columns (Figure 6). This form of PHWE is used by the company Mazza Innovations under the name PhytoClean. For example, extracts are obtained from juniper berries, rosemary, wild blueberries, parsley, algae, pecan nuts, green tea, or cocoa.

Lignins and (hemi)celluloses are another popular group of substances obtained using PHWE. These are produced by the company CH Bioforce in Finland, among others, from fresh wood that accumulates as sidestream in the production of spruce and birch.^58,59^ The products include Xylense (hemicellulose), which is used as a natural emulsifier in the cosmetics industry, as well as Lignense (sulfur-free lignin) and Cellense (cellulose). Hemicellulose^60^ and glycans^61^ are also the subject of research at the Fraunhofer IWKS in Dr. Hanstein’s working group, where they are obtained in a 0.5 L extraction vessel.

**Figure 7:**
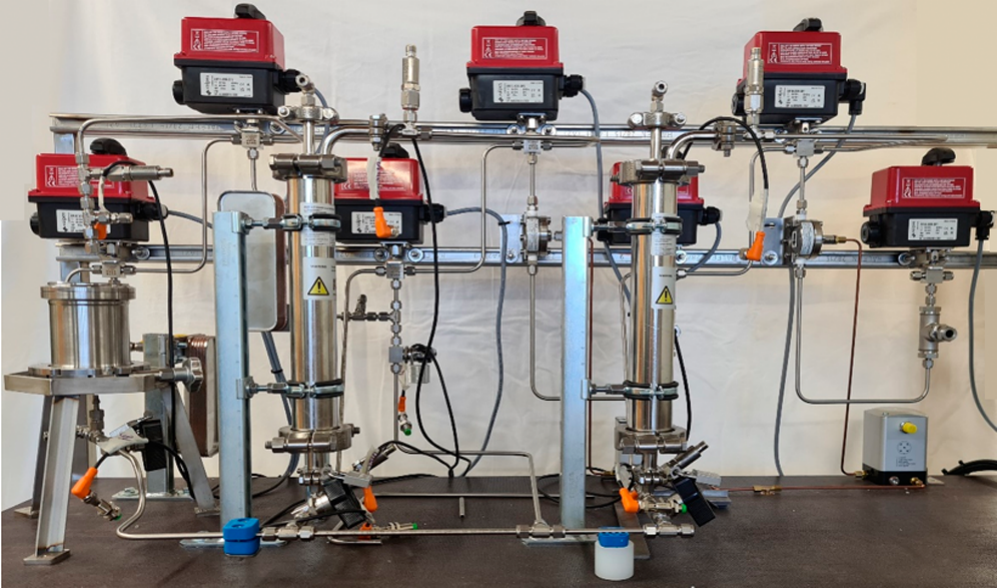
PHWE pilot plant with ultra- and nanofiltration.

For extraction and optimization at the Institute for Separation and Process Technology, a lab-scale PHWE (8 mL column) is used for screening experiments to determine the optimal temperature, particle size, and flow rate and to determine model parameters. A PHWE with a 100 mL column is then used for method optimization. An automated pilot scale PHWE with 0.5 and 1 L columns is used for advanced process control trials (Figure 7).

## Applications

As already shown in the previous chapter, PHWE can be applied to a wide range of products. To emphasize the advantages of this extraction process, the following chapter describes substance systems from the field of phytopharmaceuticals as well as food (supplements) and essential oils, which have a wide range of applications, in more detail.

### *Artemisia annua*(62)

Artemisinin, an antimalaria drug, necessitates resource-efficient and cost-effective production methods. The initial design stemmed from laboratory experiments followed by piloting on a mini-plant scale. An extensive economic feasibility study, including a benchmark reference process, compared laboratory and pilot-scale procedures, highlighting pertinent scale differences. Detailed articles elucidate unit operations such as solid–liquid extraction, liquid–liquid extraction, chromatography, and crystallization. Notably, miniaturized lab-scale experiments yield adequate data for theoretical scale-up calculations, a novel approach in academia. With the use of (P)HWE at 80 °C as an alternative extraction method for the solvent-based extraction with acetone, the space time yield (STY) could be maximized by factor of 2. In addition to that, the process time could also be reduced. Green processing targets reducing global warming potential by altering solvents and cutting solvent usage by fourfold. Optimization of liquid–liquid extraction prepurification could slash costs of goods by 80% by obviating chromatographic purification steps. Enhanced solid–liquid extractions and seamless process integration, facilitated by process modeling, promise around 40% improvements, leading to an overall operating cost reduction of approximately 25%.

### *Taxus baccata*(62,63)

Thermodynamically consistent methodologies like COSMO-RS play a pivotal role in efficient solvent screening.^64^ This research delves into a systematic and model-based strategy for process development, particularly focusing on pressurized hot water extraction while considering the potential thermal degradation of valuable compounds. In the extraction of 10-deacetylbaccatin III (10-DAB) from yew, chosen as a representative test system, water at 120 °C emerged as the optimal temperature, striking a balance between extraction yield and thermal degradation. Nearly 100% yield concerning the total 10-DAB amount was achieved within just 20 min. Model parameter determination experiments involved 1.9 g of plant material at a flow rate of 1 mL/min and a pressure of 11 bar. The physicochemical extraction model effectively assessed all experimental data, exhibiting significant conformity with simulation results (*R*^2^ = 0.958). Scale-up predictions showcased the extraction model’s utility and parameter determination accuracy. Precise predictions were attained for scale-up experiments, validating the model’s reliability. Experiments and simulation results on a 104 mL column with 22 g of yew needles mirrored the milliscale used for parameter determination. The solvent choice is pivotal, with a 40-fold reduction compared to the benchmark and 10-fold lower investment costs resulting in a remarkable 97% reduction in cost of goods. Green processing initiatives can further lead to 99.5% savings in raw material needle supply efforts.

### *Crataegus monogyna*(65)

Extracts derived from hawthorn leaves and flowers (*Crataegus monogyna* JACQ.) are categorized as “other extracts” per the European Pharmacopoeia standards, with hyperoside as the primary active compound. Traditionally, hawthorn preparations have been utilized to alleviate mild cardiac ailments.^66,67^

The commonly employed solvent-based percolation method is critically evaluated and compared against PHWE as a potential alternative to organic solvents. The study presents a systematic process design for hawthorn extraction, optimizing the solvent for traditional percolation as an ethanol–water mixture (70/30 v/v), while PHWE operates at 90 °C. Comparative analysis of extracts from various harvest batches against a commercially available product is conducted using chromatographic fingerprinting. Notably, natural batch variability is successfully integrated into the physicochemical process modeling concept for the first time.

An economic feasibility assessment demonstrates that PHWE emerges as the preferred choice not only from a technical standpoint but also economically. The study entails a systematic and model-based comparison of two different manufacturing methods for a traditionally used herbal extract. Both percolations using an ethanol–water mixture and extraction with water at 90 °C exhibit high productivity and yields, achieving a notable yield of the primary flavonoid hyperoside and the desired range of the drug extract ratio (DER).

The integration of experimental model parameter determination and robust process modeling facilitates predictive process simulation not only for the extraction of substances purified to pharmaceutical-grade but also for processing traditionally used complex extracts. These generated data sets meet regulatory requirements for quality-by-design (QbD) and Process Analytical Technology (PAT) approaches, enabling data-driven decisions for regulatory filings amidst technological advancements, market expansions, and changing regulations.

Furthermore, the economic feasibility study underscores PHWE’s ability to efficiently mitigate the financial challenges associated with solvent storage and renewal, justifying the higher investment costs for the requisite high-pressure equipment. By changing to a PHWE-based extraction process, the costs for solvent renewal are cut by 99% resulting in an overall annual cost cut for the PHWE by 50%. In addition, 20% of the personnel costs can be saved because of the shorter process time of the PHWE. The systematic application of the process engineering toolbox, encompassing physical property calculations for solvent selection, miniaturized laboratory experiments for model parameter determination, rigorous model validation, and process optimization based on cost modeling, yields substantial green processing benefits. These benefits include significant reductions in solvent consumption, water-based processing technology adoption, yield improvements, and cost of goods reductions, with potential transitions from batch to continuous operations promising substantial operational and investment cost reductions.

### *Theobroma cacao*(68)

**Figure 8:**
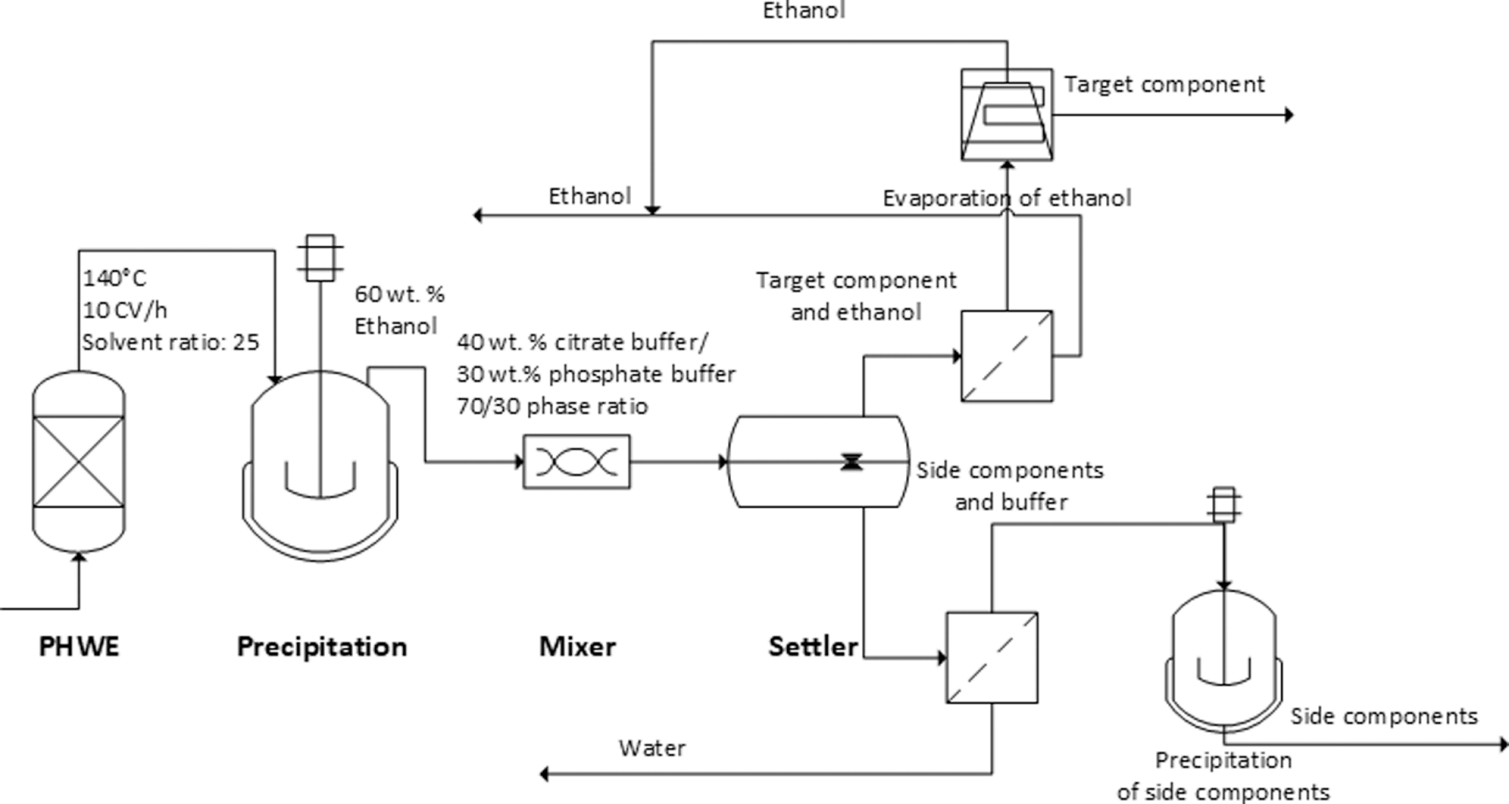
Overview of the novel process for the recovery of phenolic compounds from natural product extracts. Reprinted in part with permission from ref (68). Copyright 2022 Jensch.

Following the completion of this study, a novel process has been formulated, seamlessly integrating PHWE into a broader scheme for waste valorization. Executed in an environmentally conscious, efficient, and sustainable manner, 98–100% yield in target components was achieved by using PHWE as first extraction step. This comprehensive process comprises several key steps: hot water extraction followed by precipitation using ethanol as an antisolvent and, subsequently, liquid–liquid extraction from the resulting precipitation supernatant employing ethanol salting-out. Through meticulous integration of these unit operations, both the matrix components and the secondary plant compounds can be fully harnessed. Figure 8 illustrates the adaptable and eco-friendly process designed for waste valorization.

The innovative procedure attains yields of up to 100% within a single extraction stage, remarkably minimizing the consumption of organic solvents. Ethanol serves as the sole organic solvent utilized, and its utilization is twofold. This versatile process is adept at capturing various secondary metabolites from hot water extracts and effectively utilizes structural carbohydrates obtained from precipitation. Ethanol, renowned as a precipitant for matrix components in hot water extracts, allows for fine adjustment of its content in the light phase to align with the solubility properties of the target component, typically ranging from 50 to 80% ethanol.^69,70^

### Essential Oils^71^

**Figure 9:**
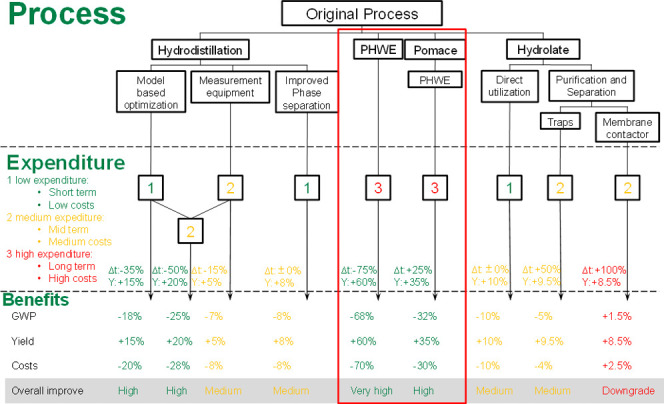
Overview and evaluation of the investigated methods and process alternatives for the production of essential oils. Reprinted in part with permission from ref (71). Copyright 2022 Jensch.

This study established efficient and scalable methods for holistic process development aimed at robust processes with cascade utilization. Leveraging digital twins, with techniques such as Process Analytical Technology, facilitates optimization of both process development and production operations, encompassing considerations of process technology and economics. The comprehensive utilization of plant raw materials and the resulting increase in value added contribute to a reduction in the global warming potential. Through the integration of the researched processes, process yield and cost of goods can be augmented by 60 and 70%, concurrently reducing GWP by 68% (Figure 9).

Roth et al.^72^ extensively discussed the creation of digital twins enlarged to essential oils,^22^ a central aspect of this study, whereas Jensch et al.^73^ examined the development process and the implementation of PAT to enable digital twins for advanced process control.^74^

### Model-Based Optimization of the PHWE Process

In the following part of the work, the PHWE process is optimized for a new material system using the digital twin. Therefore an already established process model by Sixt et al.^75^ is used.

### Digital Twin

Digital twins are virtual replicas that model physical objects or processes, facilitating real-time synchronization between the physical and virtual realms and enabling remote monitoring, control, and prediction. They can emulate various entities, from aircraft to manufacturing equipment to individuals, and are increasingly utilized across all industries and societal domains. Key applications include preventative maintenance, process monitoring, testing, and ongoing optimization.^76,77^

In process engineering, the foundation of a scalable digital twin lies in a validated physicochemical process model, which utilizes separate thermodynamics, mass transfer, reaction kinetics, and fluid mechanics, allowing transferability across scales. To function as a digital twin, the process model must be rigorously validated to accurately represent the real process, necessitating a bidirectional interface for seamless communication between the physical process and its digital counterpart. This interface must operate faster than the rate at which new states emerge in the process, enabling the implementation of model predictive control.^78,79^

The evolution of a digital twin from steady-state or dynamic process models to validated models and a digital shadow has been extensively documented.^70,74,80,81^ Model development and validation follow a prescribed process, such as the one developed by Sixt et al., and undergo rigorous testing.^64,75,82,83^ Simulation verifies the digital twin’s capability to fulfill its control objectives, preceded by process optimization using validated models and experimentally determined parameters. **Risk analysis** establishes the safe operating range, ensuring that **critical quality attributes** (CQAs) remain within specified limits, such as the DER for solid–liquid extraction. Subsequent **Process Analytical Technology** studies assess the measurement accuracy of **critical process parameters** (CPPs) determining CQAs. Simulation studies validate process stability under various disturbance scenarios while maintaining CQA limits. Upon proving controllability, the digital twin is integrated into the existing process, initially via standardized data interfaces, allowing for real-world demonstrations if data exchange and simulation occur at sufficient speeds. An adequate speed ensures that the control system can intervene in **real time**, preempting new operating states.

### Development of the Process Model

This study utilized a model developed and validated by Sixt et al.,^75^ encompassing mass transport within the extraction column through an axial dispersion model. This model accounted for both convective and dispersive flow along the axial direction of the extraction apparatus. Additionally, it considered mass transfer from solid to liquid phases, employing a pore diffusion model to elucidate mass transfer within the plant material, assuming spherical particle geometry. Desorption of the targeted components within the particles was modeled using a Langmuir model.^84,85^ The model also takes into account the thermal decomposition of the target component during the extraction process.^69^

### Determination of Process Model Parameters

Parameter determination followed the methodology outlined in Kaßing, Altenhöner, and others,^86−88^ as demonstrated by Uhlenbrock and Strube^89^ and Sixt^69^ for complex natural products extractions. Existing correlations from Altenhöner et al.^88^ were utilized to determine the axial dispersion coefficient. The maximum loading of the Langmuir model for the particles was ascertained through exhaustive percolation. Subsequently, a series of macerations were conducted to determine the equilibrium parameter Kh of the Langmuir model. Finally, quantification of pore diffusion was achieved through percolation experiments.^84,90^ The parameter for the degradation kinetic were determined as described in Sixt.^69^

### Implementation of Process Analytical Technologies

A well-established chromatography method was employed for the quantitative assessment of the target component’s content in the samples.^91^ An objective of process development within the framework of Quality by Design involves online monitoring of product quality. To this end, Fourier transform infrared spectroscopy (FTIR) was explored as a Process Analytical Technology following procedures outlined in Jensch et al.^73^ Samples from model parameter determination were measured using this method for calibration and partial least squares (PLS) model formation. A model demonstrating a strong correlation between spectrum and target component content was developed (calibration: *R*^2^ = 0.926). Moreover, previous studies indicated a robust correlation between the electrical conductivity of the extract and the extracted dry residue.^73^ Linear regression between conductivity and concentration of dry residue exhibited a commendable correlation score of *R*^2^ = 0.997. A similar procedure was executed for the system under investigation in this study, establishing a sufficiently accurate online measurement method for both the target component and dry residue.

## Results and Discussion

In the following chapter, first the results of a process control study on a PHWE extraction are shown and discussed. Afterward, the occurring effects in different extraction techniques are compared.

### Process Control Studies

The process control studies were performed to gain an insight on their influence on the yield of the extraction. Therefore, at first, one factor at a time is varied, which is followed by two studies on which multiple factors at a time are varied.

#### One Factor at a Time

To precisely understand the specific influence of each parameter, only one factor at a time (OFAT) is varied per experiment. This makes it possible to analyze the isolated effects of each parameter and avoid potential interactions between the parameters.

The variation of the adjustable parameters is done over a wide range to determine which parameters have significant effects on yield and within which range they are effective (Table 3).

**Table 3:** Parameter Variation in the OFAT Study

parameter	positive deviation	negative deviation
flow rate	44%	67%
plant material mass	25%	75%
dry residue content	69%	88%
temperature	57%	43%
target component content	87%	67%
water content	60%	140%
particle size	87%	80%

The main target parameter is the yield of the target component. Among the influencing parameters analyzed, temperature was found to have a significant effect on yield. However, an increase in temperature above a critical temperature of 130 °C leads to a negative effect on the yield.

The flow rate, on the other hand, shows a positive influence on the yield, but it becomes comparatively insignificant when temperature is varied within the range specified.

**Figure 10:**
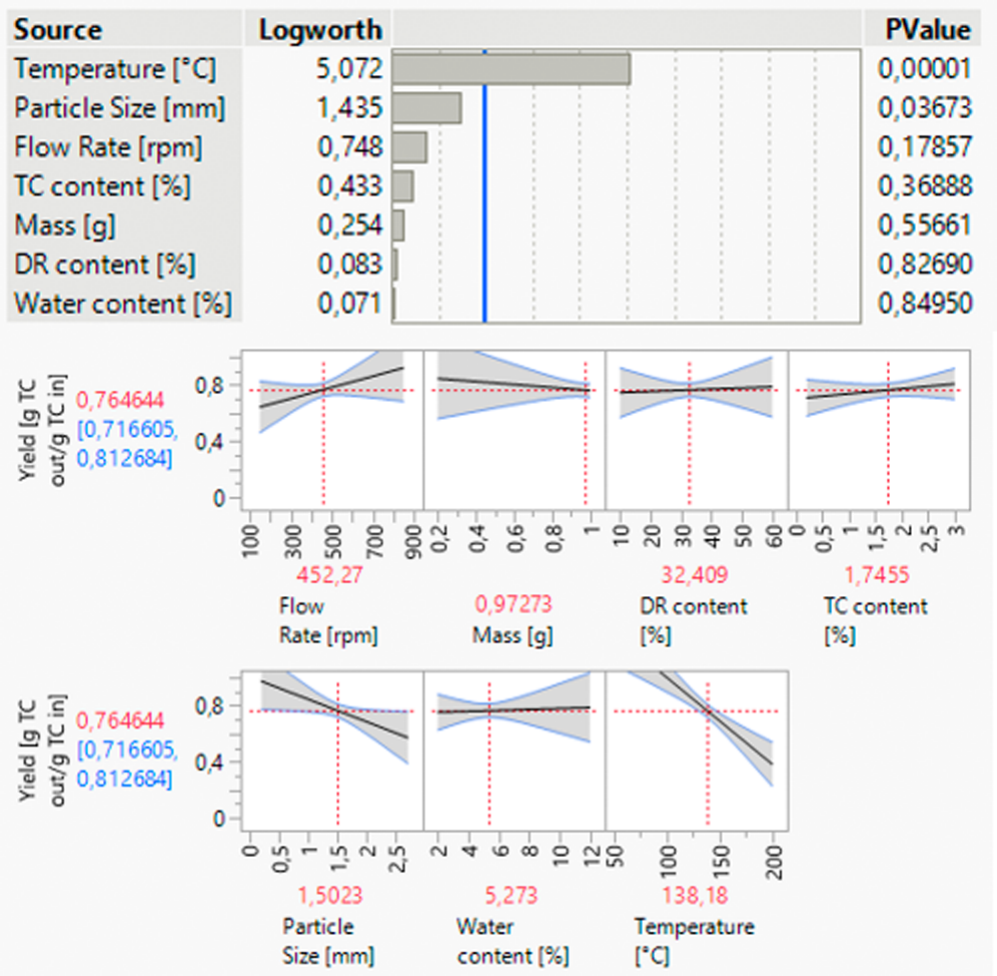
Results of the OFAT study, significance of parameters, and direction of influence.

It is also apparent that the particle size has a negative influence on the yield, as longer diffusion paths lead to a reduced yield. Within the OFAT study, the effect of particle size is compared to the effect of temperature as negligible as discussed for the effect of flow rate (Figure 10).

#### Multiple Factors at a Time: Process Parameter

In a multiple factor at a time study (MFAT), several process parameters are varied simultaneously to develop a comprehensive understanding of the process. First, parameters that can be actively adjusted in or before the process are varied, including flow rate, temperature, particle size, and the mass of plant material used (Table 4).

**Table 4:** Variation of the Process Parameters in the Process Parameter MFAT Study

parameter	positive deviation	negative deviation
flow rate	33%	33%
plant material mass	25%	25%
temperature	14%	14%
particle size	33%	33%

**Figure 11:**
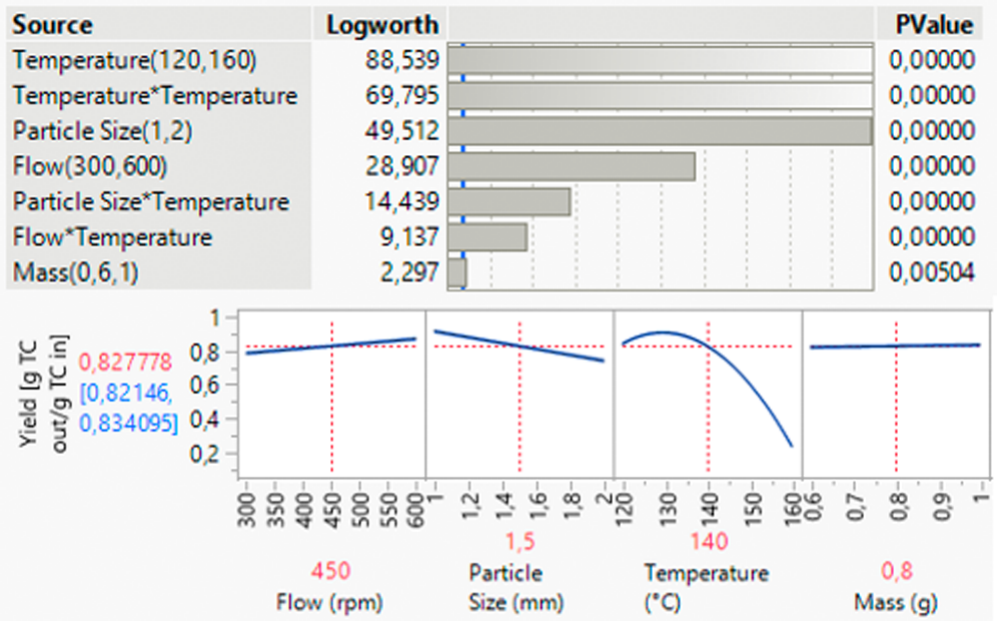
Results of the MFAT study of the process parameters, significance of parameters, and direction of influence.

The yield of a PHWE process is influenced by various factors, with temperature exerting the greatest influence. However, it is important to note that temperature is not only linear but quadratic and affects the effects of other variables such as particle size and flow rate illustrated by the interaction coefficients (Figure 11). As the temperature increases, the yield decreases sharply because of the thermal decomposition of the target component above 130 °C.

The particle size also plays an important role, with smaller particles being advantageous as they enable shorter diffusion paths and thus increase the efficiency of the process (Figure 11).

The flow rate is another important factor that influences the yield. A higher flow rate leads to a higher yield as it promotes mass transfer. The mass has no significant influence on the yield (Figure 11).

#### Multiple Factors at a Time: Material Parameter

In this MFAT analysis, material parameters are changed that can be influenced such as the used mass of the plant material and the particle size as well as material parameters that cannot be influenced like dry residue and the proportion of the target component in the plant material (Table 5). This study is done to get an insight of the influence of the material parameters on the yield of the process without being overlaid by process parameters, e.g., temperature.

**Table 5:** Variation of the Material Parameters in the Material Parameter MFAT Study

parameter	positive deviation	negative deviation
target component content	33%	33%
plant material mass	25%	25%
dry residue content	25%	38%
particle size	33%	33%

**Figure 12:**
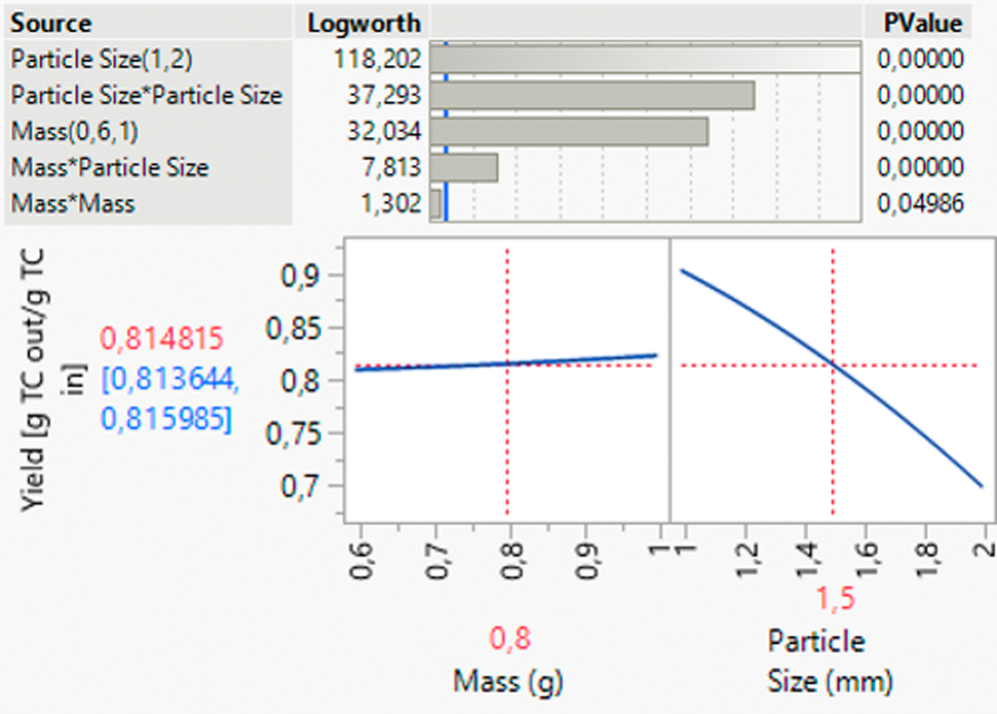
Results of the MFAT study of the material parameters, significance of parameters, and direction of influence.

When considering the material parameters, the yield of the PHWE process is significantly influenced by the particle size, with smaller particles enabling a higher mass transfer due to their shorter diffusion paths. In addition, the particle size does not have a linear but a quadratic effect on the yield and also interacts with the mass of the plant material used. These interactions illustrate the complexity of process optimization and the variety of influencing factors (Figure 12).

The mass of the material used also plays a significant role in the yield. A higher mass of plant material used generally leads to a higher yield, as more starting material is available for the process (Figure 12). The dry residue and target component content show no influence on the yield.

The results generated using the digital twin in the MFAT studies are statistically analyzed with the statistical software JMP using linear regression to determine not only the influence of each parameter but also the ideal process parameters. The ideal parameters for the material system shown are a temperature of 130 °C, a flow rate of 600 rpm that is equal to 1.5 mL/min, a particle size of 1 mm, and a quantity of plant material of 1 g to achieve the highest yield.

### Comparison of Extraction Methods and Effects

**Figure 13:**
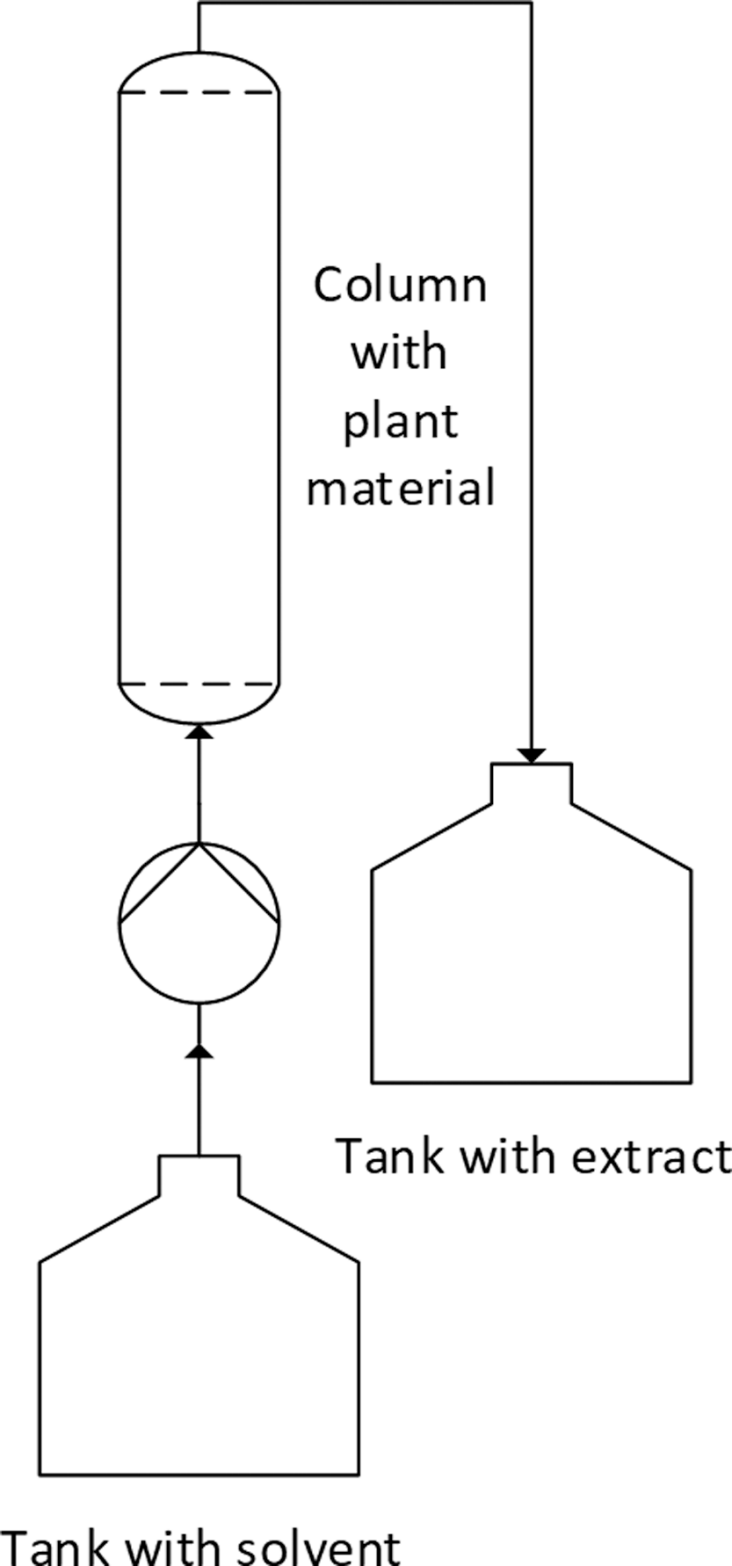
Example process for organic solvent based plant extraction as percolation.

**Figure 14:**
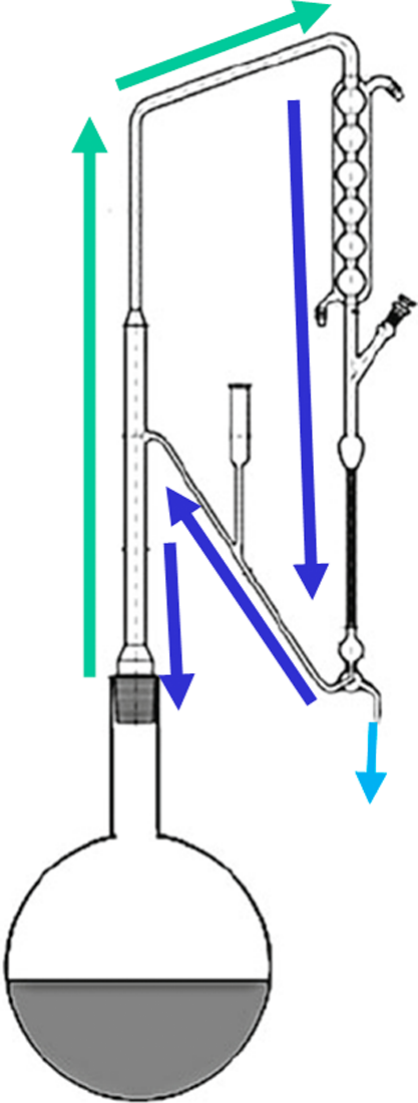
Example process for hydrodistillation of essential oils according to European Pharmacopoeia. Reprinted in part with permission from ref (94). Copyright 2009 European Pharmacopoeia.

When comparing PHWE with other extraction methods such as classic solid–liquid percolation with organic solvents (Figure 13) and hydrodistillation (Figure 14), it is noticeable that PHWE is less selective than the other two extraction methods. PHWE tends to be a total extraction, whereas hydrodistillation is selective for (essential) oils and fatty acids. The selectivity of extraction with organic solvents is in between. Here, selectivity can be adjusted via the polarity of the solvent but is not as selective as hydrodistillation for essential oils. By using the PHWE, the dielectric constant can be adjusted from values of 40 to 60 via alternating the temperature, which makes it a suitable extraction method for polyphenols. The polarity of an organic solvent based extraction with ethanol–water solutions can be adjusted via the ratio of both solvents in ranges of 24,5 to 80,1 for the dielectric constant, extracting polyphenols as well as sugars. The values of the dielectric constant for hydrodistillation only vary in the range of 5 to 15; therefore, this method is selective for (essential) oils. Depending on how high the selectivity of the extraction method is, more or fewer purification steps are then required to concentrate or isolate the target component.^92^ The advantages of PHWE are that, in contrast to extraction with organic solvents, it is water-based, green, kosher, and halal as well as faster than hydrodistillation, such higher throughput, and productivity, i.e., smaller plants and less solvent amounts. In addition, the water used can be purified and recovered with the aid of low-energy technologies like ultrafiltration or nanofiltration. The advantage of using organic solvents to extract plant material is that, in contrast to hydrodistillation and PHWE, low temperatures are used and the system does not have to be pressurized. The disadvantages here are the more complex purification and recovery of the solvent used. When using hydrodistillation, the essential oil separates again after condensation of the vapor above the hydrolate, but there is a residual solubility of the oil in the hydrolate, which means that parts of it are lost,^93^ i.e., about 0.35%.

**Figure 15:**
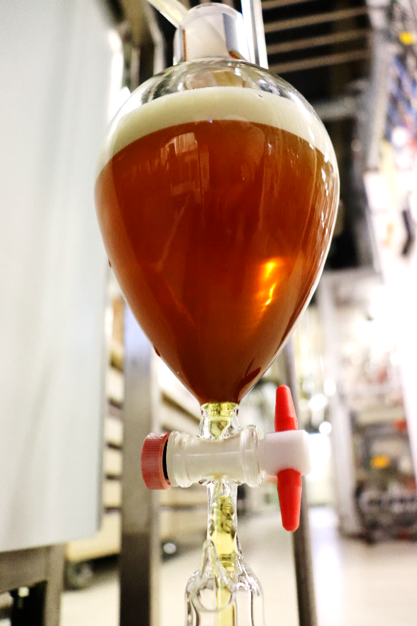
PHWE extract of chamomile in a separation funnel with no visible essential oil.

**Figure 16:**
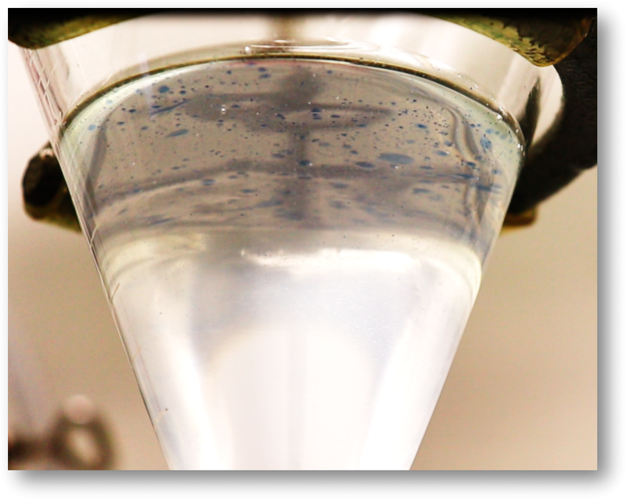
Hydrolate and visible essential oil of the hydrodistillated PHWE extract.

**Figure 17:**
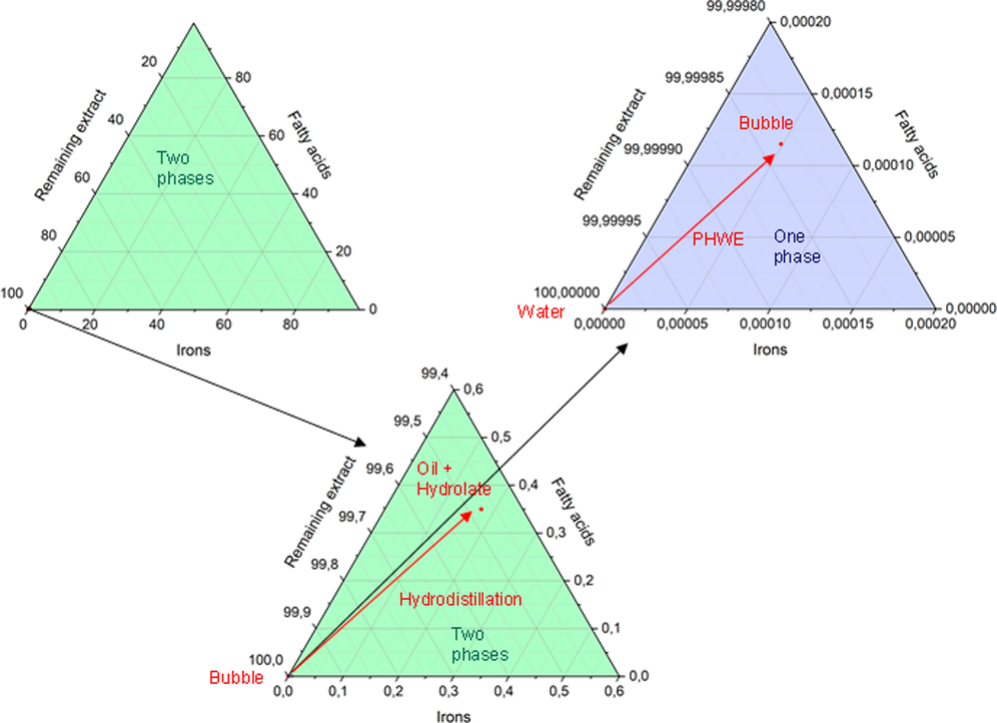
Phase diagrams of the hydrodistillation and PHWE process for the extraction of essential oils from *Iris pallida*.

If PHWE is used as an alternative extraction method to hydrodistillation, it is noticeable that the essential oil does not settle on the surface after the extract has cooled (Figure 15), as is the case with extraction using organic solvents. However, if a PHWE extract is subsequently hydrodistilled, the essential oil becomes visible above the hydrolate again (Figure 16). To get to the process comprehension of these phenomena, the phase equilibrium diagrams of the individual extraction methods are analyzed: Figure 17 shows that the composition of the PHWE extract is in the single-phase region, which means that the essential oil is dissolved. However, the composition of the hydrolate is in the two-phase region, whereby the solubility of the essential oil in water is exceeded and the oil and water phases separate.

**Figure 18:**
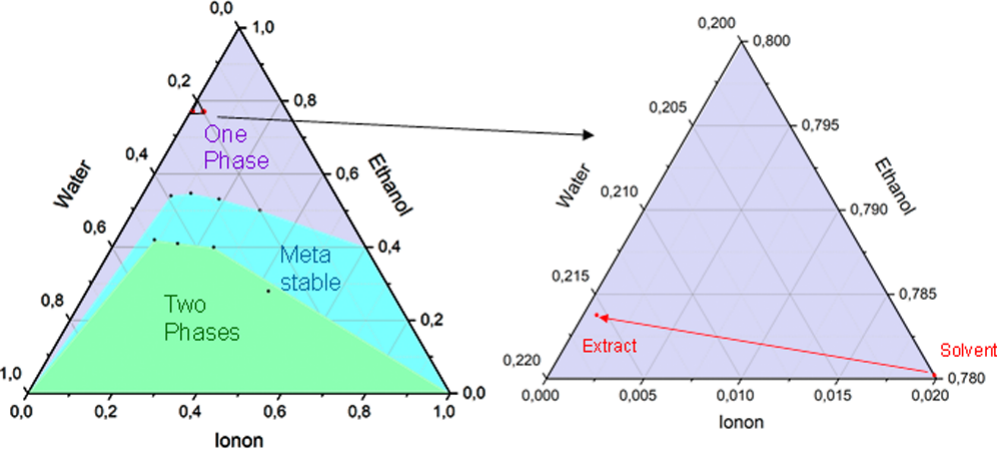
Phase diagrams of the ethanol–water based process for the extraction of essential oils from *Iris pallida*.

The phase equilibrium diagram of the extraction with ethanol–water mixtures (Figure 18) shows a significantly larger one-phase range than that of the purely aqueous extraction. However, the composition occurring in the extraction used here is also in the single-phase region of the phase equilibrium. When looking at the general composition of other mixtures of water, ethanol, and essential oils, such as those used in the perfume industry, it is noticeable that they are all in the two-phase region.^95,96^ One reason for this is that the proportion of essential oils in plants used to produce fragrances ranges from 0.2 mg/g plant material (*Rosa x damascena*,^97^*Pelargonium* sp.,^98,99^*Cistus ladanifer*^100^) to 0.7 mg/g plant material (*Iris pallida* Lam.),^97^ and another is that phase separation is not desirable in the common products such as eau de toilette or perfume.^95,96^

## Conclusions

The demand of traditional natural products, be they remedies, cosmetics, dietary supplements or even agrochemicals, known for their significant benefits, has been steadily increasing and making a significant contribution to global health for some 20 years, especially given the recent tightening of regulatory standards for drug safety and advances in manufacturing technology, as well as the fact that the health insurance system does not allow reimbursement^101^ without evidence-based studies and reauthorization.

**Figure 19:**
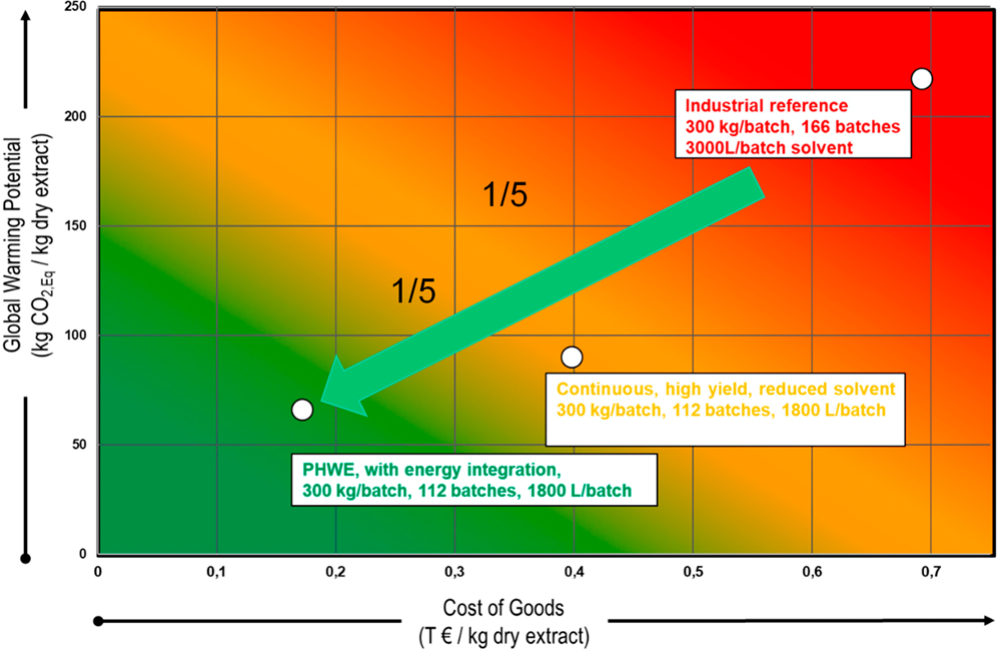
CoG and GWP reduction potential of the PHWE with energy integration and water recycling. Reprinted with permission from ref (102). Copyright 2020 Schmidt.

Innovative manufacturing technologies, such as water-based processing utilizing pressurized hot water extraction followed by concentration with membrane technologies like nanofiltration and ultrafiltration, hold considerable potential to achieve climate neutrality targets and realize cost savings, thereby enhancing competitiveness in global markets. Substantial reductions, up to a factor of 5, are attainable through modern process design (Figure 19).

The adoption of a Quality by Design approach, advocated and mandated by regulatory authorities, ensures drug quality assurance and enhancement. Transitioning from traditional batch-wise operations to continuous processes significantly reduces resource requirements.

The incorporation of digitalization and Industry 4.0 methods, including machine learning and artificial intelligence, empowers traditional natural product extraction to effectively compete in present and future markets in nutrition additives, cosmetics, and agrochemicals, which are as well extremely under cost of goods pressure as well as open for technology changes due to regulatory options.

PHWE is an innovative extraction method that is not only kosher and halal but also water-based and sustainable. Based on renewable raw materials and resilient plants, it offers an environmentally friendly solution. Through energy integration, PHWE is not only sustainable but also energy efficient. By integrating ultrafiltration and nanofiltration, water recovery is also possible, which leads to a further reduction in environmental pollution.

**Figure 20:**
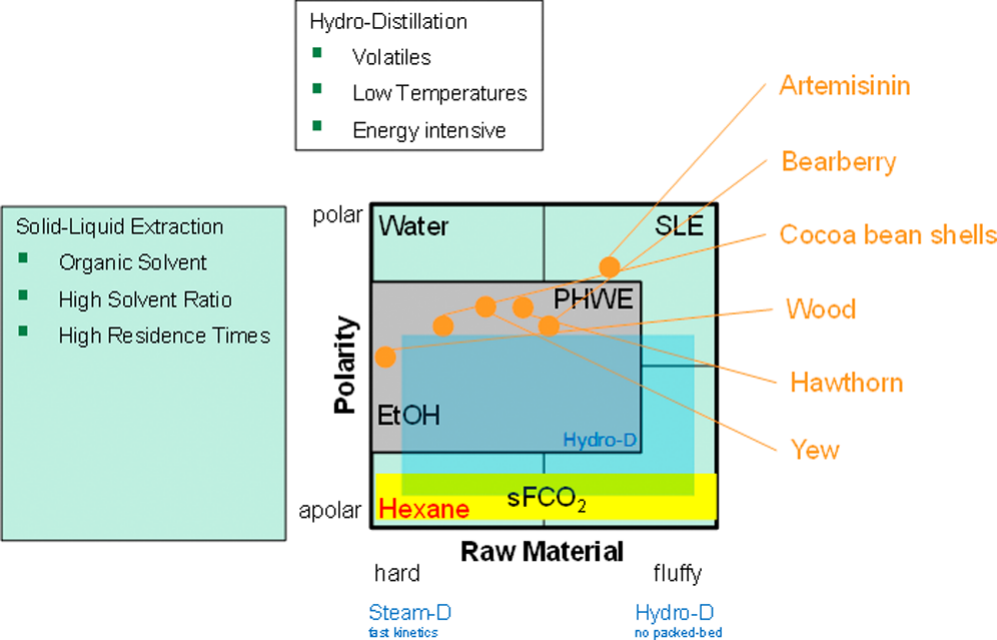
Applications of PHWE in comparison with other extraction methods and solvents.

By using PHWE, as a summary, any of the substance systems shown in the technology portfolio of Figure 20 can be extracted in a water-based, environmentally friendly manner and without organic solvents. Other benefits of the extraction of natural resources with PHWE are the production process that also leads to green and natural products at low cost of goods as well as a low global warming potential due to efficient energy integration.

## ABBREVIATIONS

PHWE: pressurized hot water extraction
GWP: global warming potential
CoG: cost of goods
STY: space time yield
10-DAB: 10-deacetylbaccatin
DER: drug extract ratio
QbD: quality by design
PAT: process analytical technology
CQA: critical quality attributes
CPP: critical process parameter
FTIR: Fourier transformed infrared spectroscopy
PLS: partial least-squares regression
OFAT: one factor at a time
MFA: multiple factor at a time
